# Citizen-led sampling to monitor phosphate levels in freshwater environments using a simple paper microfluidic device

**DOI:** 10.1371/journal.pone.0260102

**Published:** 2021-12-09

**Authors:** Samantha Richardson, Alexander Iles, Jeanette M. Rotchell, Tim Charlson, Annabel Hanson, Mark Lorch, Nicole Pamme

**Affiliations:** 1 Department of Chemistry and Biochemistry, University of Hull, Hull, United Kingdom; 2 Department of Biological and Marine Sciences, University of Hull, Hull, United Kingdom; 3 Pocklington Canal Amenity Society, Pocklington, United Kingdom; 4 East Riding of Yorkshire Council, Beverley, United Kingdom; University of Illinois at Chicago, UNITED STATES

## Abstract

Contamination of waterways is of increasing concern, with recent studies demonstrating elevated levels of antibiotics, antidepressants, household, agricultural and industrial chemicals in freshwater systems. Thus, there is a growing demand for methods to rapidly and conveniently monitor contaminants in waterways. Here we demonstrate how a combination of paper microfluidic devices and handheld mobile technology can be used by citizen scientists to carry out a sustained water monitoring campaign. We have developed a paper-based analytical device and a 3 minute sampling workflow that requires no more than a container, a test device and a smartphone app. The contaminant measured in these pilots are phosphates, detectable down to 3 mg L^-1^. Together these allow volunteers to successfully carry out cost-effective, high frequency, phosphate monitoring over an extended geographies and periods.

## Introduction

The European Commission Water Framework Directive (WFD) sets out legislation to ensure that all waterbodies across Europe achieve a ‘good’ ecological status [1, 2]. In 2016, 86% of UK rivers failed to reach this status; of the assessed water bodies, 55% featured excess levels of phosphate, resulting in failure to reach the desired ‘good’ status [3]. Nutrient levels, however, are not constant, they vary widely spatially and temporally, and patterns are often missed due to infrequent measurement [4]. Therefore, to improve water quality and better understand whether nutrient levels meet the aims of the WFD, robust and frequent monitoring of water quality is vital to safeguard supplies and to manage the health of aquatic ecosystems [5, 6].

Current routine water quality monitoring generally relies on established analytical methods such as high performance liquid chromatography (HPLC) or UV/vis spectroscopy, whereby a trained expert will go out into the field to collect samples which are then taken back to a laboratory, prepared and analysed [7]. These techniques are costly and time consuming and thus monitoring is carried out at low frequency and low spatial resolution [5, 8]. Routine monitoring is often only conducted on a monthly basis at best, with the sampling points along a river limited by time, resources and constraints in territory [8, 9]. A fuller understanding of water quality and contaminant dynamics, including sources and behaviour of contaminants are often lacking, hindering cost-effective and targeted environmental management [9]. A low-cost, easy to use method will facilitate better quantification of trends and pressures, underpin predictive modelling and provide the foundation for robust and cost-effective management of the aquatic environment.

High frequency sensing could be achieved with simple and low-cost devices operated by citizens or lightly trained agents. Data can then be uploaded to a cloud to build a picture of a larger area that is not easily obtainable by sending expert scientists into the field. According to the literature, some on-site systems have been developed as well as some autonomous systems for passive monitoring [10, 11]. However, currently such devices are relatively expensive, require several setup steps or require expertise to perform the manual steps in the workflow, such as calibration or sample preparation, making them unsuitable for regular low cost monitoring through volunteer-led sampling campaigns.

Dip tests or microfluidic Paper-based Analytical Devices, PADs, offer a promising alternative for on-site analysis with sampling methods simple enough to be completed by a non-expert [12, 13]. Devices can be readily produced by patterning commercially available hydrophilic cellulose filter paper with a hydrophobic material such as wax [14, 15] to create channels and reaction zones within the paper matrix. Reagents can be preloaded and stored in dry form on the devices. Fluid is transported into and through the paper by capillary forces. Meanwhile, via a smartphone app, users could conveniently photograph results from the PADs and upload these images along with records of the time and location of the measurements. Here, we set out to develop a PAD and app for truly simple on-site monitoring of contaminants by members of the general public. By taking such an approach we aim to allow regular and frequent on-site measurements by volunteer groups across a wide area not previously achieved.

The chosen analyte, phosphate, is an important example of a potential freshwater pollutant [16]. Phosphates are essential nutrients present in freshwater environments at low concentrations (0.005 to 0.05 mg L^-1^) [17]. However, it is well documented that aquatic levels are often artificially increased by run-off from agricultural and domestic activities [17–19]. Excessive amounts of phosphates, *i*.*e*. in the milligram per litre range, can lead to eutrophication; the rapid growth of algae [20]. In severe cases thick algal blooms reduce oxygen levels in water bodies and stop sunlight reaching beyond the surface of the water; in the most serious cases, the decomposition of the algae can lead to build-up of harmful toxins [19–21]. Problems associated with phosphate induced eutrophication include reduced fish populations, excessive death of fish during summer period, and changed composition of aquatic species in affected water bodies [22].

Detection of orthophosphates, the main form of bioavailable phosphate linked to eutrophication, [23] is typically performed using UV/vis spectroscopy via formation of the phosphomolybdenum blue (PMB) complex [24–26]. Occasionally an ion exchange chromatography method is also employed [27]. Both methods require laboratory equipment and expertise to operate. Phosphate test strips are commercially available from some providers, including Hach (UK); these rely on a colour change that can be compared to a colour chart. As the colour change is usually very subtle, it can be easily misinterpreted, leading to incorrect recording of phosphate levels. Other kits mostly require the mixing of reagents, typically nitric acid, making them less suitable for use by an untrained operator for on-site analysis.

Jayawardane *et al*. reported a paper-based microfluidic device for phosphate analysis from water samples [28]. They created two reaction zones to separately store the reagents needed for the PMB reaction. Sulfuric acid at 6.6 M was employed, which required careful optimisation to avoid damage to the paper by hydrolysis of the cellulose [29]. A Teflon sheet had to be placed between the two layers and sealed by lamination. Before use, the device had to be cut open and the Teflon sheet removed. A working range of 0.6–30 mg L^-1^ and limits of detection and quantitation of 0.15 and 0.48 mg L^-1^, respectively, were obtained. These are environmentally relevant levels. However, samples were added via a pipette and results were recorded via a flatbed scanner after 10 min incubation. The detection zones were 3 mm in diameter and hence rather small for visual inspection. The reported paper device was thus not usable by volunteers working in the field.

In contrast to the previously reported work, we set out to develop a simple to operate phosphate detection device with colorimetric readout that requires minimum input from the operator, yields a result within a few minutes that can be captured via a smartphone camera, thus avoiding the use of dedicated detection equipment. We show that such a system can be used by lightly trained volunteers to collect a significant data set, and this therefore is an appropriate tool to deploy across a wide area to elucidate patterns in spatial and temporal variations of waterway contamination pressures.

## Experimental

### Design and fabrication of the paper devices

Details of reagent preparation and stock solutions are given in **SI1 in S1 File**. Paper-based devices were wax printed on Whatman Grade 1 filter as outlined in **SI2 in S1 File**. The wax pattern was printed in two areas that were folded on top of each other to form an upper and lower reagent zone (**S2 Fig in S1 File**). The design used for field-based sampling, featured eight 10 mm circles enclosed with a rectangular wax box to reduce leaking in case of poor alignment between the upper and lower paper layers. These devices featured six detection zones (n = 6) as well as two negative control zones. At low analytle concentrations, any colour pigmentation in the water could potenially lead to a false positve result. Therefore, two of the eight circular zones were not loaded with any reagents, thus acting as blanks. This design also had a four-digit code so that individual devices could be identified.

**Fig 1:**
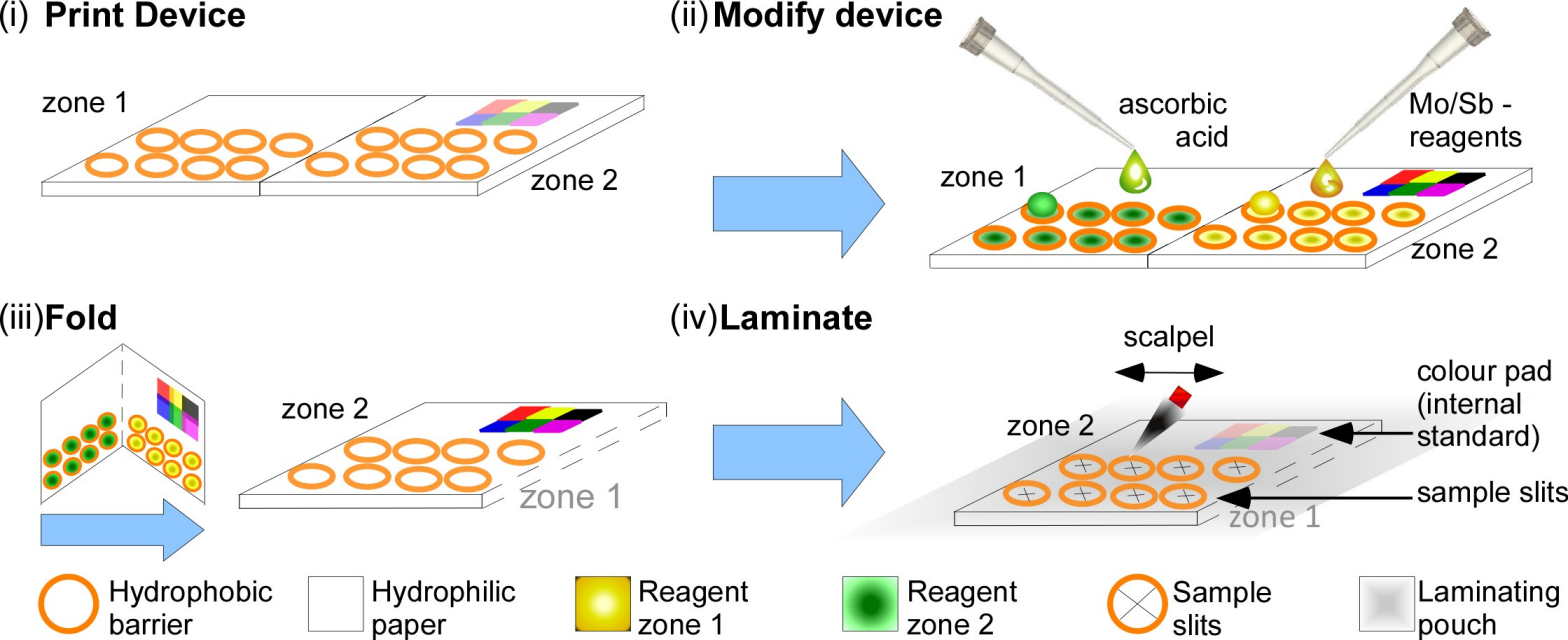
Production of PAD. (i) Circular reaction zones were wax printed onto filter paper. (ii) Ascorbic acid solution was added to each circle in zone 1, Mo/Sb reagent to each circle in zone 2. After drying, the paper was backfolded to align the sample zones. (iii) Devices were sealed by lamination to encase the reagents and prevent contamination. (iv) Slits were cut into the back of the device (zone 1) to allow for sample entry.

The process of loading reagents and sealing the devices is shown in **Fig 1**. Reagents were pipetted onto the respective circular zones and left to dry. The paper was backfolded and then sealed by lamination to maintain alignment and enclose the deposited reagents. More specifically, 5 μL of the ascorbic acid reagent and 5 μL of molybdate/antimony reagent were pipetted onto zone 1 and zone 2, respectively. The 5 μL volume was chosen as it was sufficient to cover the 10 mm zone effectively whilst leaving no excess liquid on the surface thus minimising drying time. The devices were allowed to air dry for about 30 min and then further dried overnight (-20°C) to ensure complete evaporation of water, before being backfolded and laminated at 80°C with matte finish 150 micron pouches (Lyreco, UK). Two slits in an x-pattern were cut with a scalpel into the back of the device to allow for water entry.

### Sampling and data collection

**Fig 2:**
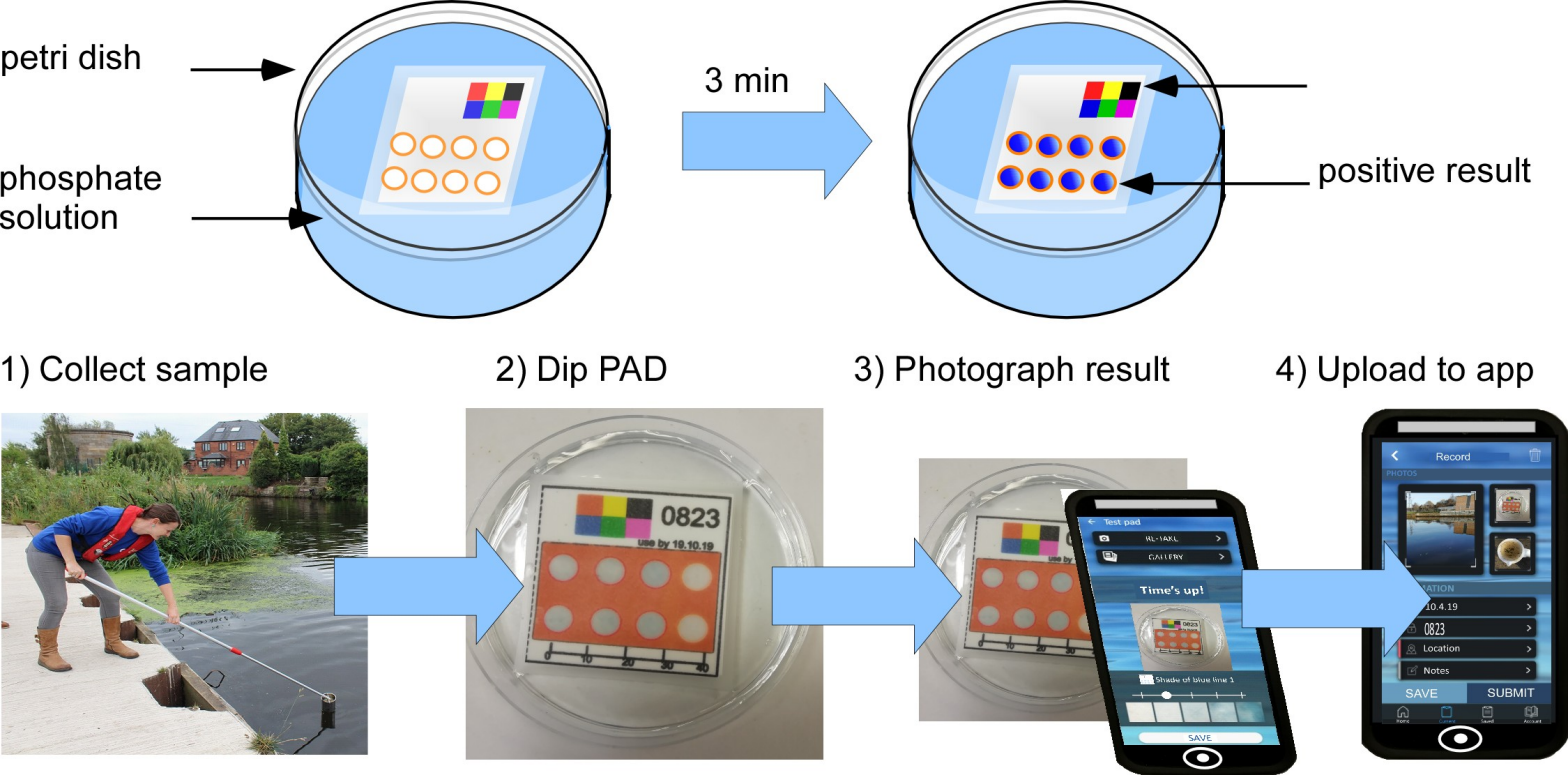
**(a)** The laboratory analysis workflow involved the paper device being placed into a dish with 20 mL aqueous sample. The sample entered through the slits in the back of the devices. After 3 min incubation, the formation of the blue colour on the upward facing side of the device was captured. **(b)** The workflow for volunteer sampling involved the collection of a water sample from a river and an aliquot being placed into a container. The paper device was dropped into the sample, the same as in the laboratory, and after 3 min, a photograph of the result was taken and uploaded via the RiverDip app.

Development of the analysis workflow was a key factor in the design of the device. The laboratory-based analysis workflow needed to be as similar as possible to the real-world analysis to achieve a device fit-for-purpose and to validate the sampling methodology. Thus, we decided to use a simple dip method (**Fig 2**). In the laboratory, the device was placed in a dish containing approximately 20 mL of sample, with the front side facing upwards. Water soaks into the device until the cellulose in the reaction zone is saturated, the sample components are left to react with the pre-deposited reagents. Following optimisation, a short incubation time of 3 min was found to be sufficient to allow for a stable colour to form. After this time, the image was captured using either a flatbed scanner (CanoScan LiDE 220) or a smartphone camera (Huawei P smart). In the field, the same protocol was followed, the volunteers would dip the device on a sample of freshwater and the image of the device was collected using the volunteers’ own phones and the custom-developed RiverDIP app. Image analysis was carried out with ImageJ freeware as detailed in **SI3 in S1 File**. Benchmarking against the gold standard UV/vis spectrophotometry method was undertaken as described in **SI4 in S1 File**.

**Fig 3:**
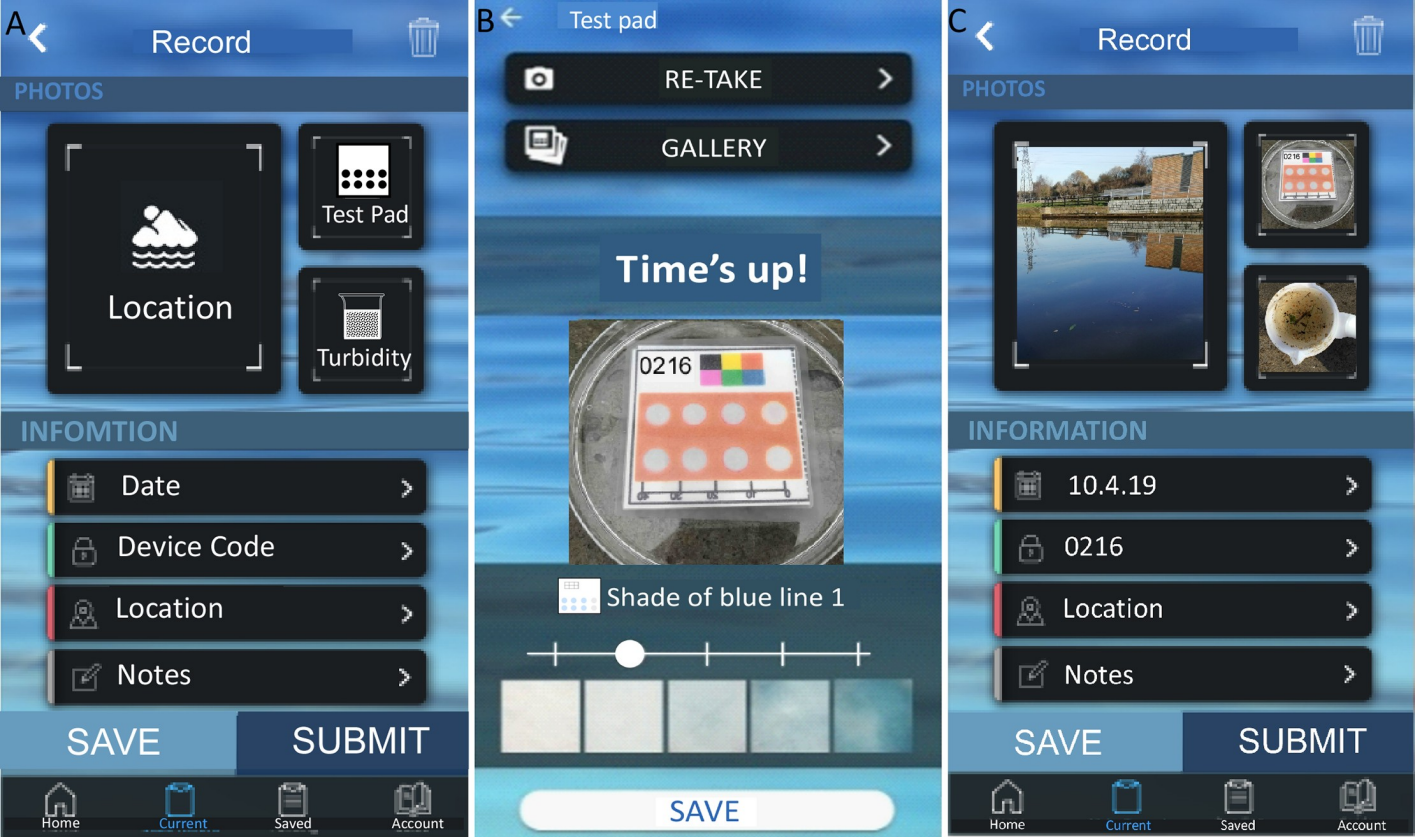
The RiverDip app was custom-developed to record test results in the field, with GPS location and time alongside photographs of the water quality and the surroundings. (a) The ‘record navigation page’ shows the data that needs to be completed for each sampling. (b) ‘Capturing test PAD result’ will start a 3-min timer, after which an image can be captured and uploaded. Volunteers can compare the result to a colour intensity scale bar and select the colour intensity that matches their results the closest. (c) Screenshot showing a completed result ready for submission with date, time, record code and location and images of the PAD result, location and water quality.

RiverDip (**Fig 3**) was developed in collaboration with Natural Apptitude Ltd (UK), a company specialising in app developing including for Citizen Science projects. The app is available via the Apple or Android Appstore. After logging on, the user is required to read safety information and confirm understanding. The user can then select to carry out a measurement, *i*.*e*. ‘start a new record’. The user will take a photo of the paper microfluidic device from within the app, following running down of a 3 min timer to ensure the test result is taken after the required incubation time. Furthermore, the user can upload photographs of the sampling location and water turbidity. In addition, the app records date, time and location of the sampling, with the user manually inputting the device code and name of the water body sampled. Users also give their own interpretation of the results and water turbidity using a colour sliding scale. They can then leave free-form comments if they wish. The data is uploaded onto a server and can be retrieved later for quantification of phosphate levels using image analysis software.

### Study areas

Engagement with volunteers was covered under ethics agreement FEC_2018_26, reviewed by Faculty of Science and Engineering Ethics committee, University of Hull. Informed written consent was obtained from individual volunteers prior to participation in any sampling actives.

Volunteer sampling was carried out across the North Sea region in 2019 to 2021, with sampling across the UK, The Netherlands, Belgium and Germany. Alongside this wider sampling campaign some smaller sites were sampled more frequently in particular along a section of the Pocklington Canal (between GPS locations 53°54’57.5"N 0°47’01.5"W and 53°52’32.7"N 0°56’07.4"W) within the Humber catchment (UK). This is a typical example of a managed lowland waterway heavily influenced by rural agricultural activity, and, despite its status as a site of special scientific interest (SSSI), has a history of nutrient enrichment [30]. The canal is approximately 15 km long, 8 km of this being navigable. Sampling was undertaken along a 7 km stretch of the canal including both navigable and non-navigable sections.

Comparison of PADs to the gold standard UV/vis analysis method was carried out using samples from the Pocklington Canal and River Aire. Both are located within the Humber catchment, however, the River Aire represented a water system heavily influenced by urban activity in comparison to the Pocklington Canal. This gave a range of water samples to study the effect of sample matrix on the colorimetric reaction chemistry.

## Results and discussion

### Optimisation of reagents and method

The standard method for phosphate analysis via the phosphomolybdenum blue (PMB) reaction, [26] as well as the previously reported paper-based method, [28] rely on sulfuric acid to provide the acidity needed to minimise auto-reduction of the ammonium molybdate reagent. However, it is well documented that sulfuric acid readily hydrolyses cellulose, [29] the main component of the filter paper material used in paper microfluidic devices. Therefore, instead of sulfuric acid we chose to use p-toluenesulfonic acid (TsOH), a non-oxidising solid acid with pKa -1.34 that allowed the required low pH to be achieved whilst avoiding paper hydrolysis.

A key factor when designing the sampling method was to have a short incubation period to avoid testing the volunteers’ patience when taking measurements in the field. The PMB reaction is time dependant, a heteropoly acid complex is reduced over time to form the brightly coloured product [26, 31–33]. Many variations of this reaction have been reported with incubation times ranging from 90 s to several hours [26]. As a starting point, we used concentrations of 0.01 M Mo reagent together with 0.01 M ascorbic acid as the reducing agent, similar to methods previously reported with incubation times of up to 10 min [34–38]. Antimony tartrate is commonly added when using ascorbic acid as the reducing agent to improve the rate of reduction and to remove the need to heat the reaction [25]. For the here reported work, 0.6 mM antimony tartrate was used.

**Fig 4:**
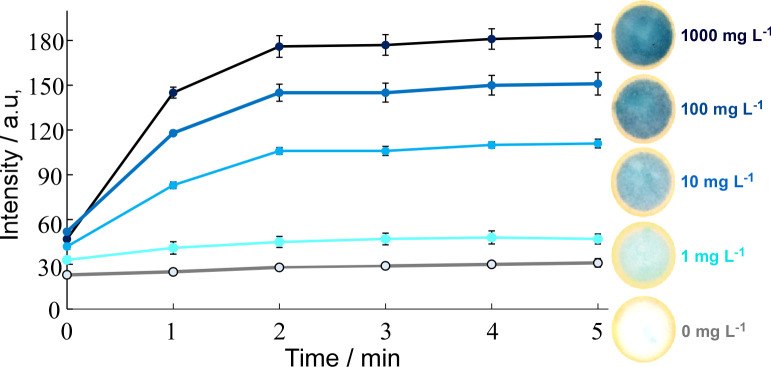
Formation of the blue PMB complex over time at different phosphate concentrations. The colour intensity was found to increase for up to 2 min and then plateau (n = 3).

The incubation time required to obtain a stable readout of the blue coloured PMB complex needed to be optimized. Ideally this process would take less than 5 min and be visible to the human eye. A series of experiments was performed with 5 μL of molybdate/antimony reagent (0.01 M Mo and 0.6 mM Sb in 2 M TsOH) and 5 μL of ascorbic acid (0.01 M) for phosphate concentrations of 0, 10, 100 and 1000 mg L^**-1**^. The reactions were carried out under laboratory conditions at room temperature (25°C) and images captured using the flatbed scanner every minute and analysed with ImageJ. The results are plotted in **Fig 4**. The colour was found to rapidly develop in the first minute of the reaction for all concentrations of phosphate solutions other than the 1 mg L^**-1**^ solution. The colour intensity then, more slowly, increased further up to 2 min at which point it began to plateau. At 3 min, the colour was found to be stable across the sample zone. Based on these findings, 3 min was deemed the ideal length for the volunteer-based dip tests; long enough for the colour to develop and become stable; yet still short enough for work with volunteers out in the field.

To ensure maximum sensitivity from the device whilst minimising auto-reduction of the molybdate complex, a careful balance between [Mo(VI)] and [H+] must be achieved. Optimisation of these conditions is described in **SI5 in S1 File**.

### Limits of detection and quantification

Following the optimisation experiments, calibration curves for to determine limits of detection (LOD) and limits of quantification (LOQ) were generated from devices prepared by pipetting of 5 μL of molybdate/antimony reagent ([Mo(VI)] = 0.01 M / [antimony] = 0.6 mM) in 2 M TsOH into zone 1 and 5 μL of 0.01 M ascorbic acid into zone 2 prior to lamination. This method was used to obtain calibration data (**SI6 in S1 File**) giving an LOD and LOQ of 3 mg L^-1^ and 8 mg L^-1^, respectively. Eutrophication can occur when phosphate levels exceed 0.1 mg L^-1^, [39] however levels much higher were sometimes detected by the UK environment agency in recent years during routine water quality monitoring. Water quality records from 2018 show available phosphorous levels between 0.001–20 mg L^-1^. With unplanned sampling recording levels into the 100’s mg L^-1^ [40]. The PAD devices we have developed here feature a relatively high limit of detection. Despite this the devices can still be used to screen large areas by taking a semi-quantitative approach. This can provide very useful information about a river system and highlight areas that may have excessive phosphate levels, which can then be further investigated.

### Stability

To enable volunteer-based sampling campaigns, PADs need to be stable for a reasonable period of time to allow devices to be distributed and used in a realistic time frame. This was addressed to some extent by storing the two reagents separately on different sites on the paper devices on the two reaction zones which were back- folded to minimise contact (see **Fig 1**). Whilst this approach increases the lifetime of the reagents, it does not prevent the auto-reduction of the molybdenum complex, which is typically controlled by low pH, however not eliminated completely.

To assess the viability of distributing the phosphate PADs to volunteer groups, we studied the performance of the devices following storage under different conditions over a 4 week period. Devices were prepared as stated in section 2.1 and then stored in the dark in a closed box, at room temperature with and without silica gel desiccant sachet, to elucidate how moisture affected long-term storage. Devices were also stored in a domestic fridge (4°C) and freezer (-20°C). Device performance was tested on day 0, day 7 and day 28 by performing the 3 min dip test with a 10 mg L^-1^ PO_4_^3-^ solution. The results are shown in **SI7 in S1 File**. It was found that devices stored in the freezer were stable for the full length of the period investigated and this approach was therefore used for long term storage of the devices. The more important aspect of the stability test was that over a shorter period of time, *i*.*e*. 1 week, devices remained stable when stored in ambient conditions. After 1 week of storage, devices stored at room temperature yielded the same intensity compared to those used straight away when tested with 10 mg L^-1^ phosphate solution. Thus the devices are stable for long enough to allow for batch manufacturing, distribution via postal services, cold storage on arrival and subsequent use by volunteer groups.

### Inferences

To ensure the PADs can be used to accurately analyse the levels of phosphate in river water, it was important to ensure cross-reaction with other species in a water sample matrix does not either interfere with the colour readout or give a false reading. Silicates (SiO_4_^4-^) in the water system are reported as the main interferent in the PMB reaction, they form heteropoly acid complexes with 12-MPA [41]. This is seen particularly at high pH [37, 42, 43] and temperatures [42]. To investigate the potential for silicate interference when using the PADs for environmental measurements, 10 mg L^-1^ phosphate solutions were doped with silicate at a range of concentrations (100–1000 mg L^-1^) and the observed intensities were compared to a typical intensity value achieved when measuring a solution of 10 mg L^-1^ PO_4_^3-^ only. The results as shown in S8.1 and S8.2 **Figs in S1 File** demonstrate that the silicate reaction occurs over significantly longer timescales. Therefore, over the 3 min incubation time period, silicate present in the water course does not interfere with the phosphate measurements. We also investigated whether silicates would produce a false positive result when no phosphate was present in the solution. At 1,000 mg L^-1^ silicate and an incubation period of 3 min the silicate readout was equivalent to a blank sample, thus demonstrating that silicates in the water system will not produce a false positive result.

Whilst no interference from silicates was found, many other species could be present in a water sample that may interfere with the PMB reaction. To investigate this, Milli-Q water and canal water samples were spiked with phosphate. Samples from the Pocklington Canal (Yorkshire, UK) were tested, the canal is a water source with high calcium carbonate (100’s mg L^-1^) and chloride levels (up to 100 mg L^-1^) as well as many other water soluble ions in sub-mg L^-1^ levels [40]. Both water samples yielded results with no significant difference to a blank sample (t_stat_ = 5.127, t_crit_ = 5.849 at α = 0.05; n = 8 for a two-tailed t-test). Therefore, it was assumed both samples contained no detectable levels of phosphate. The canal samples were then spiked with phosphate, in the range of 0–10 mg L^-1^ and 0–1000 mg L^-1^, and compared to the same concentrations spiked into Milli-Q water (S8.3 **Fig in S1 File**), demonstrating that there was no inference from the sample matrix.

### River water measurements

**Fig 5:**
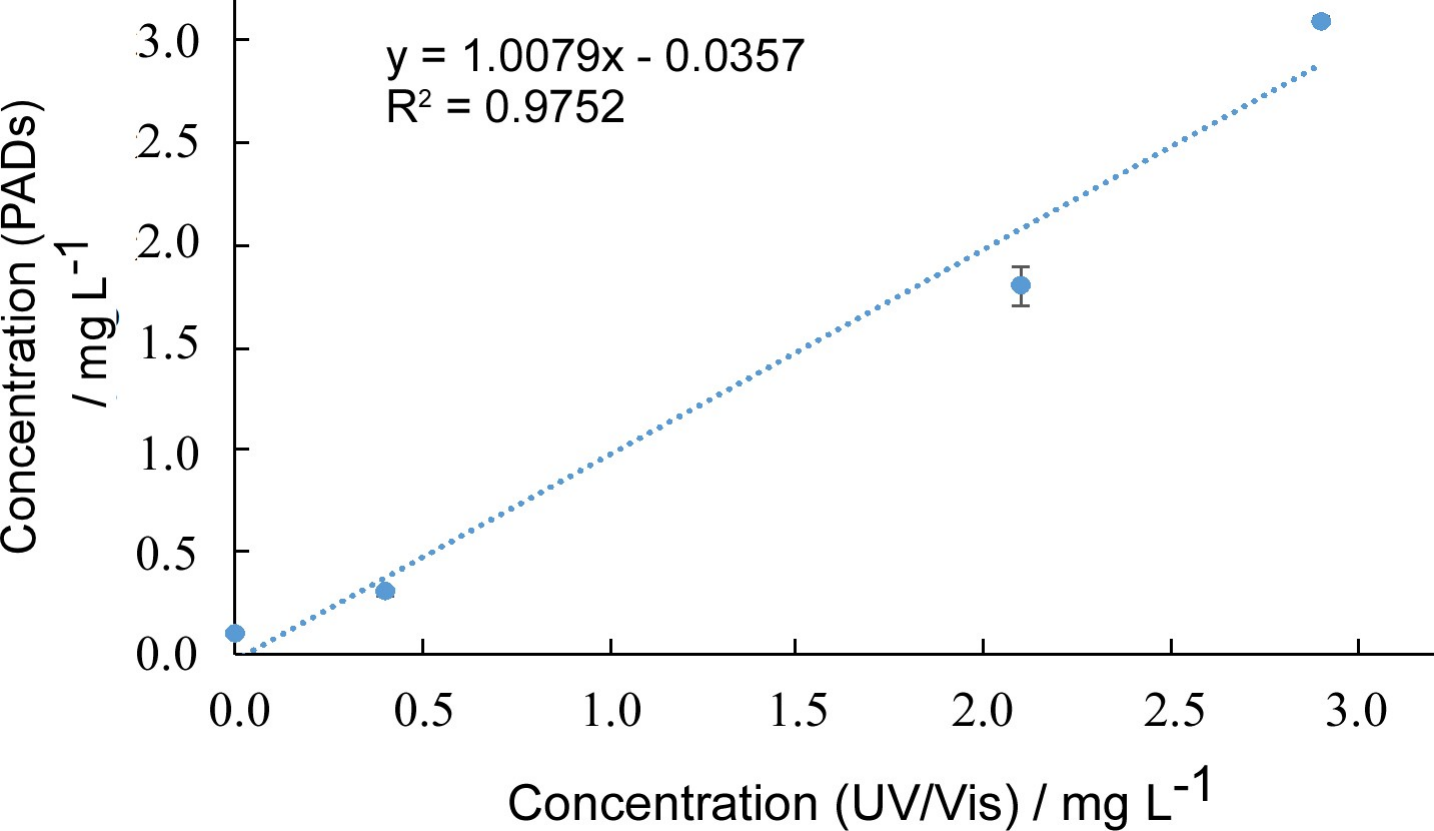
Comparison of PO43- levels in freshwater samples detected using UV/vis spectroscopy via PMB reaction (n = 3) and modified PMB method on the paper microfluidic device with flatbed scanner image capture (n = 6).

Next, the paper PADs were tested with samples collected from the River Aire and Pocklington Canal (both within Yorkshire, UK). Water samples were stored at 4°C prior to laboratory analysis. The results obtained from the PADs and the industry standard UV/vis spectroscopy method [44] are shown in **Fig 5**. As can be seen, both methods gave comparable results. Whilst the traditional spectroscopy method gives more accurate results, both would yield the same phosphate level (to 1 s.f.). The PADs however can be used in the field and could be employed to rapidly gather large amounts of semi-quantitative data.

**Fig 6:**
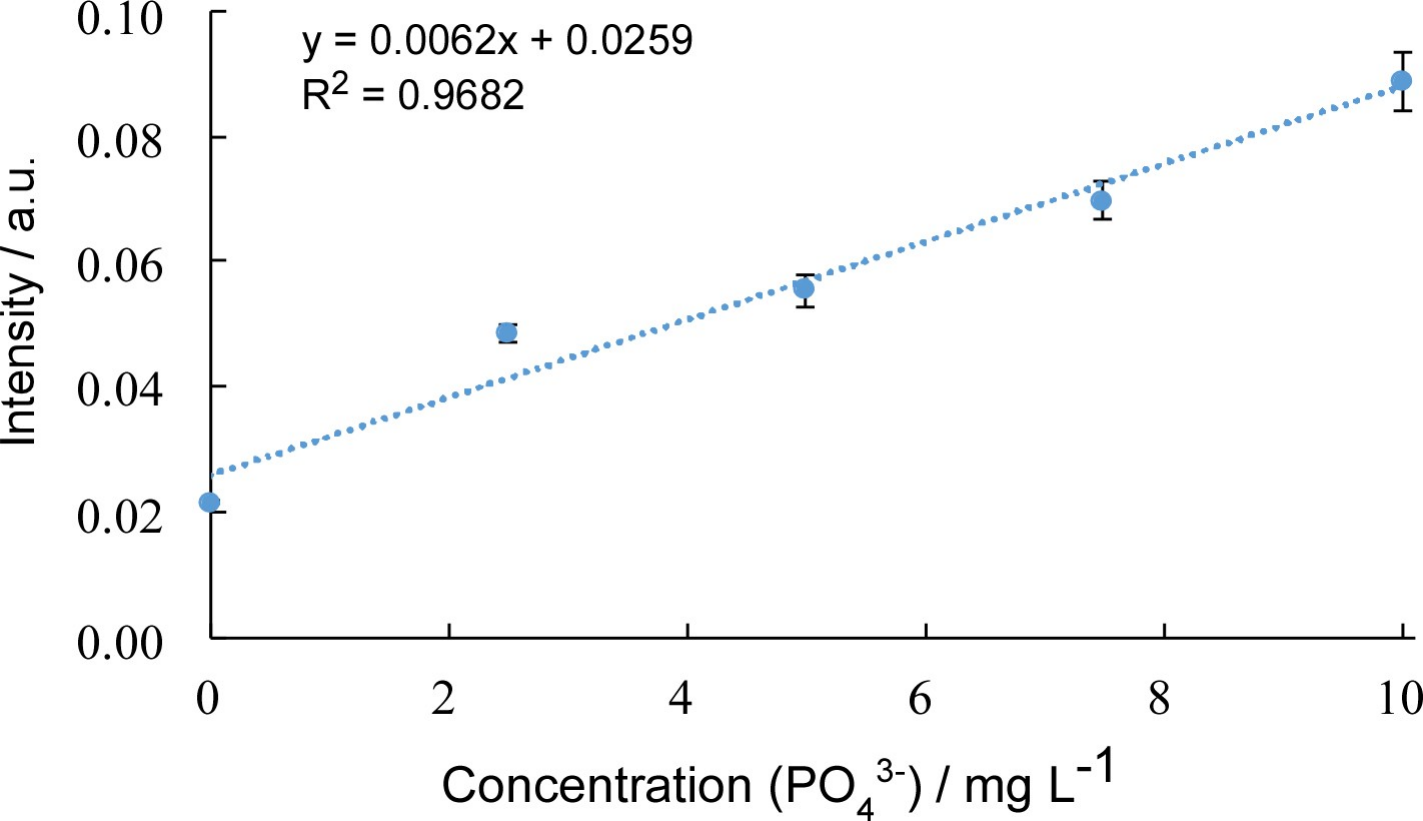
Typical calibration curve obtained with images captured from a smartphone camera. The obtained colour development in the sample reaction zones were standardised against both the internal standard (blue square) and the negative controls to account for interferences from varying light conditions and background colour from the water sample (n = 6).

When carrying out field sampling it is important to account for changes in environmental conditions such as lighting, colouration or turbidity of the water samples and camera to camera differences. At low analytle concentrations, as would be expected, it is possible that any colour pigmentation in the water could lead to a false positive result. To address this, two negative control zones were introduced into devices used in the field. These zones were left blank so that the colour intenisty read from them would relate only to the colour of the water sample (**S9 Fig in S1 File**). Average relative intensity (ARI) for the real freshwater samples were calculated from the average pixel intensity (AI) of the reaction zones and the average pixel intensity of the blank zones (Eq 1). Using this method, a calibration curve was constructed (**Fig 6**) that would be suitable for the analysis of field sampling results.


$$ARI=\frac{{AI}_{reaction\;zone}-{AI}_{blank\;zone}}{{AI}_{ref.\;sq.}}$$ (Eq 1)

To ensure comparability between results obtained using different image capture methods, we determined phosphate levels in freshwater samples when captured using a flatbed scanner (laboratory-based detector) and a smartphone (field-based detector) (**S9 Table in S1 File**). We found that both image capture methods yielded the same phosphate concentration with all samples tested. Using a scanner to capture the image gave a more reproducible result as the lighting was constant, however, results captured using a smartphone, in a less controlled environment, still produced the same overall result. This shows the viability of these devices for volunteer-led sampling campaign to capture large amounts of data out in the field to reliably indicate levels of phosphate.

Following this optimisation, the devices were tested with volunteer groups from the local Canal and River Trust (CRT), who manage and maintain water courses such as the Pocklington Canal, UK. Group leaders were trained to carry out sampling, capture and upload the data to the RiverDIP app before leading sampling sessions across the Humber catchment area. This was further extended to volunteers across the North Sea Region specifically in Belgium, the Netherlands and Germany, who undertook sampling independently after initial training. During 2019 and 2021 over 350 samples were collected by volunteers across the North Sea Region.

**Fig 7:**
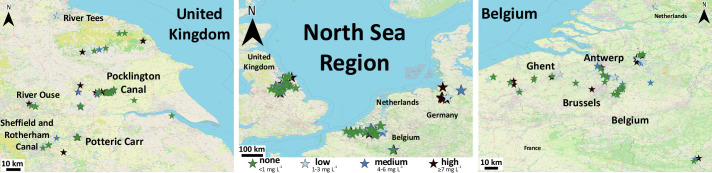
Data collected via citizen-led sampling sessions across the North Sea region presented for public dissemination via a Google map embedded in the project page website (https://riverdip.com/map). Since Google’s maps are copyrighted they cannot be displayed here, therefore the figure shown (produced using QGIS Desktop 3.16.10 software republished under a CC BY license with permission from QGIS) is similar but not identical to the map used to update volunteers on the progress of the monitoring campaign. Throughout 2019 and 2021 over 300 samples were collected by volunteer across the region. The location of the marker represents the GPS location of sampling, the colour of the marker represents the obtained phosphate level (none <1 mg L^-1^, low 1–3 mg L^-1^, medium 4–6 mg L^-1^ and high ≥ 7 mg L^-1^). Each marker links to other details captured on the RiverDip app at the time of sampling; including the user’s own interpretation of the phosphate level, a photo to record water turbidity, and any notes made during the sampling.

By using volunteers to perform sampling across a large geographical area and with frequent measurement, large amounts of data could be gathered, showing trends and pressures not otherwise seen by traditional analysis. One way to represent field data is via a map ‘pins’ for each sampling point. The devices are capable high resolution measurements (as shown in the calibration curve in **Fig 6**), however to simplify presentation of the data on the public facing map we bracketed the results into four phosphate levels (none <1 mg L^-1^, low 1–3 mg L^-1^, medium 4–6 mg L^-1^ and high ≥ 7 mg L^-1^) each represented by a different coloured pin on the map. Such data could be used to display results to a public audience. An example of this is shown in **Fig 7** representing all the successful tests carried out in this period, n = 332 Here, we display results gathered from volunteers across the North Sea region. In certain areas volunteers engaged in routine sampling across longer time periods. This allowed use to detect seasonal variations in phosphate levels along a 16 km stretch of the Pocklington Canal (**S10 Fig in S1 File**). This demonstrates that the PADs, app and workflow are a cost-effective method to monitor waterways across large areas and the resulting data can be used to detect seasonal trends and contaminant pressures not seen with traditional analysis methods.

## Conclusions

We have demonstrated a simple to use paper microfluidic device suitable for on-site field analysis by lightly trained volunteers. The devices return semi-quantitative readings, with a limit of detection of 3 mg L^-1^ of phosphate levels in freshwater samples in just 3 minutes. Results obtained from using these methods were comparable to those obtained using the laboratory gold standard method of UV/vis analysis. The paper devices record a colour change which was captured using a smartphone camera and uploaded via the custom-designed RiverDip app for further analysis. The image analysis method accounts for variations in environmental lighting conditions and turbidity in the sample. We also confirmed there was no interference from silicates nor other constituents of in a freshwater sample.

Devices can be manufactured and stored for at least 4 weeks, allowing distribution via mail. Field tests have confirmed that the PAD devices can be used by volunteers to gather the data with little input from experts. This approach has great potential for environmental data to be gathered across wide geographical areas, thus potentially providing freshwater quality readings with high spatial and temporal resolution to better monitor and respond to pressures.

## Supporting information

S1 File (DOCX)
