# Supplementary material for: Citizen-led sampling to monitor phosphate levels in freshwater environments using a simple paper microfluidic device
Source: PLoS One. 2021 Dec 9;16(12):e0260102. doi: 10.1371/journal.pone.0260102 (PMC8659362; doi:10.1371/journal.pone.0260102)
Supplement: S1 File — (DOCX) [file pone.0260102.s001.docx]

Electronic Supplementary Information (ESI)

Citizen-led sampling to monitor phosphate levels in canal and river water using simple paper microfluidic device

Samantha Richardson,^a^ Alexander Iles,^a^ Jeanette M. Rotchell,^b^ Mark Lorch,*^a^ and Nicole Pamme *^a^

a. Department of Chemistry and Biochemistry, University of Hull, Cottingham Road, Hull, HU6 7RX, UK.
b. Department of Biological and Marine Sciences, University of Hull, Cottingham Road, Hull, HU6 7RX, UK.

[1.	Reagents	2](#_Toc69392041)

[2.	Paper Device preparation and Designs	2](#_Toc69392042)

[3.	Image Analysis	4](#_Toc69392043)

[4.	Benchmarking with UV/vis spectrophotometry	5](#_Toc69392044)

[5.	Optimisation of Molybdate and Toluenesulfonic acid concentrations	5](#_Toc69392045)

[6.	Laboratory-based calibration under optimal conditions	6](#_Toc69392046)

[7.	Stability	7](#_Toc69392047)

[8.	Interferences	7](#_Toc69392048)

[9.	Field results	10](#_Toc69392049)

[10.	References	10](#_Toc69392050)

# 1. Reagents

A stock solution of 1,000 mg L^-1^ containing sodium dihydrogen orthophosphate (BDH, UK) was used to prepare working standards in the range from 0 to 1,000 mg L^-1^. Molybdate/antimony reagent for the detection of orthophosphate was prepared from 0.01 M ammonium heptamolybdate tetrahydrate (Fisher Scientific, UK) and 0.6 mM potassium antimony(III) tartrate hydrate (99 %, Fisher Scientific, UK) dissolved in 2 M p-toluenesulfonic acid (TsOH) (Fisher Scientific, UK). The reducing reagent consisted of 0.01 M L-ascorbic acid (AA) (Fisher Scientific, UK). A stock solution of 1,000 mg L^-1^ sodium orthosilicate pentahydrate (Fisher Scientific, UK) was used to prepare working standards to test for silicate interference. Reagents were prepared in plastic vessels to avoid potential silicate contamination from glassware. Stock solutions were stored in a refrigerator and kept for no longer than one week; ascorbic acid solutions were freshly prepared each day.

# 2. Paper Device preparation and Designs

Paper-based devices were designed using AutoCAD 2016 software and printed onto Whatman Grade 1 filter paper using a Xerox ColorQube 8570 wax printer with ColourQube wax inks as described by Carrilho *et al*.^1^^,^ ^2^ Adapting a method from Peters *et al.**^3^* the wax printed sheets were passed through a laminator (Fellows, model Callisto A4) three times at 125 °C. This allowed the wax to melt and flow through the porous paper to form a hydrophobic barrier throughout the entire paper thickness.

The wax pattern was printed in two areas that were folded on top of each other to form an upper and lower reagent zone. **Design 1** (Figure S2a) featured eight pairs of circles, 10 mm diameter and 8 mm centre to centre distance, printed with 0.7 mm line width in orange wax. The orange colour allowed for a good contrast to the blue colour developed during the PMB reaction. As these devices were to be used by volunteers in the field, it was important to have a detection zone that can be easily visualised and to this end we settled on 10 mm diameter. Each device also included a set of six coloured squares as internal standard to account for changes in lighting condtions and camera to camera differences during image analysis. A further design change was introduced, **Design 2** (Figure S2b) whereby the eight circles were enclosed with a rectangular wax box to reduce leaking in case of poor alignment between the upper and lower paper layers. At low analytle concentrations, any colour pigmentation in the water could potenially lead to a false positve result. Therefore, two of the eight circular zones were not loaded with any reagents to act as blanks for in-the-field devices. Any colour in these zones would origniate from the colour of the water sample. These devices thus featured six detection zones (n = 6) and two negative control zones, the latter allowing for correction due to water colouration or turbidity. A four digit code (marked XXXX in figure S2b) was also included so that individual devices could be identified. Design 2 was rolled out for use with volunteers.

**(a)**

**(b)**

**Figure S2**: **(a)** Design 1 with wax circle dimension before heating. **(b)** Design 2 with circular openings in a wax area to minimise leaking of reagents due to poor alignment of the reagent zones.

# 3. Image Analysis

Images, whether captured in the laboratory or in the field, were analysed with ImageJ freeware (National Institutes of Health, USA) (figure S3). Akin to a method published by Boehle *et al.*,^4^ the .jpg images were first inverted. The image was then split into the three separate colour channels (RED, GREEN and BLUE) and only the RED channel was used for further image analysis, as this was found to give the largest difference for measurements of varying sample concentrations. The area enclosed by the orange wax barrier was analysed by measuring the mean grey pixel intensity to give an average pixel intensity for each circle. Data was obtained from the six to eight circles on the paper device; outliers were removed using the Dixon Q-test^5^. To minimise effects from variations in environmental conditions, matte laminating pouches, instead of glossy pouches, were used; and an internal coloured square was included in the PAD design (see figure 1). Average relative intensity (ARI) was obtained by dividing the average pixel intensity (AI) for the individual circles by the average pixel intensity of the blue reference square (equation S1).

$ARI= \frac{{AI}_{spot}}{{AI}_{ref. sq.}}$	eq S1.

**(c)**

**(b)**

**(a)**

**Figure S3**: ImageJ analysis process for individual paper device **(a)** The captured .jpg image was inverted and split into the RGB channels. **(b)** The first step in the image analysis process where the captured image was inverted. **(c)** The RED channel only was used for image analysis of each reaction circle and the blue printed square.

# 4. Benchmarking with UV/vis spectrophotometry

Results from the PADs were validated by comparison to the industry standard UV/vis spectrophotometry method.^6^ This was performed in triplicate. The molybdate/antimony reagent was prepared by adding 5 mL H_2_SO_4_ [5 M] to 0.5 mL potassium antimonyl tartrate [0.4 mM], 1.5 mL ammonium molybdate [0.3 M] and 3 mL ascorbic acid [0.1 M]. This solution could be stored for up to 4 h in a dark container. To perform the test, 1 mL of this reagent solution was added to 4 mL of phosphate sample or river water sample and vortexed for 30 s. After an incubation period of 20 min, absorbance was measured at 880 nm on a UV/vis spectrometer (Jenway 6705). A reagent blank was used as reference solution.

5. Optimisation of Molybdate and Toluenesulfonic acid concentrations

To ensure maximum sensitivity from the device whilst minimising auto-reduction of the molybdate complex, a careful balance between [Mo(VI)] and [H+] must be achieved. There are various forms of agents used to reduce the 12-MPA, the most common being ascorbic acid and tin chloride. Of these, ascorbic acid was the preferred choice due to the rapid formation of a stable product which is unaffected by chloride interference.(26) The ammonium molybdate complex used in the PMB reaction can undergo direct reduction if the acidity is too low; typically reactions are performed between pH 0.5-1 to minimise this. However, increasing the concentration of H^+^ too far will inhibit the reaction, reducing the sensitivity of the device. A range of TsOH concentrations were screened (0-4 M, pH 5.3 - 1.4) whilst all other reagent concentrations and volumes remained consistent at 0.01 M Mo / Sb 0.6 mM and 0.01 M ascorbic acid, with 5 µL added to each reagent zone. Each device was dipped into a 10 mg L^-1^ phosphate solution for 3 min and an image captured using the flatbed scanner (figure S5a). The maximum reproducible sensitivity was achieved when using 2 M TsOH, therefore this was chosen as the concentration for further experiments. Removing TsOH from the system did not prevent the PMB reaction from occurring, however the results became erratic, seen by the high level of error produced from one reaction to the next. The lack of acidity allowed several Mo(VI) species to form, rather than just having the MoO42- species present as needed for the 12-MPA complex to form efficiently. Increasing the acidity favours the formation of the 12-MPA species, thus better controlling the reaction, as seen at 1 M and 2 M TsOH. However, above 2 M TsOH concentration, the formation of the final colour product was supressed, seen as a significant reduction in intensity when using 3 M or 4 M acid. Similar trends have been previously demonstrated by Nagul et al. when using excessively high acidities.^(26)^

**Figure S5.1**: Intensity of the blue colour formed from PMB complex after 3 min incubation in 10 mg L^-1^ PO_4_^3-^ solution with varying TsOH concentrations (n = 8).

With a fixed acid concentration of 2 M TsOH, the Mo(VI) concentration was then studied between 0.005 M and 0.02 M to maximise sensitivity without increasing the signal generated by a blank water sample. As can be seen from figure S5b, increasing the concentration of molybdenum in the system increased the amount of the PMB complex produced, thus increasing the colour intensity. A concentration of 0.01 M was shown to give the maximum response whilst still minimising background colour in the blank. Above this value, the intensity of the blank dramatically increased, showing that the direct reduction was no longer controlled. Although there was little difference between the intensity of 0.005 M and 0.010 M Mo(VI), lower concentrations would potentially lead to inconsistent results as shown in figure S5b, where the lower concertation produced a patchy response due to an insufficient quantity of Mo(VI) present in the reaction zone.

**Figure S5.2**: Photographs of devices and blue colour intensity measurement when incubated for 3 min with varying [Mo (VI)] concentrations. Devices were tested in both 10 mg L^-1^ PO_4_^3-^ solution and a blank of Milli-Q water (n = 8).

# 6. Laboratory-based calibration under optimal conditions

**Figure S.6** shows a typical calibration curve obtained under laboratory conditions, images were captured using a flatbed scanner (CanoScan LiDE 220) after 3 min incubation in sample solution. This was used when optimising and comparing laboratory-based methods to field results.

**Figure S6**: Linear relationship between concentration and intensity between 0 and 10 mg L^-1^ PO_4_^3-^ using the optimised device preparation and 3 min incubation time. Images were captured using a flatbed scanner and analysed with ImageJ to compare the intensity of the internal standard to the reaction zone. LOD (PO_4_^3-^) = 3 mg L^-1^ and LOQ (PO_4_^3-^) = 8 mg L^-1^ (n = 8).

# 7. Stability

Laminated devices with slits already cut into the back of the device were stored under different conditions to determine stabilty over a one month period.

**Table S*7*** Average intensity after 3 min incubation in 10 mg L^-1^ phosphate solution for devices stored under different conditions on day 0, day 7 and day 28 after production (n = 8).

| Storage method | Intensity / a.u. | | |
| --- | --- | --- | --- |
|  | **Day 0** | **Day 7** | **Day 28** |
| Freezer (-20 °C) | 0.25 ± 0.004 | 0.25 ± 0.030 | 0.25 ± 0.010 |
| Fridge (4 °C) | 0.25 ± 0.004 | 0.25 ± 0.020 | 0.32 ± 0.020 |
| Cupboard (20 °C) | 0.25 ± 0.004 | 0.25 ± 0.020 | 0.30 ± 0.020 |
| Cupboard (desiccant, 20 °C) | 0.25 ± 0.004 | 0.41 ± 0.080 | 0.41 ± 0.040 |

# 8. Interferences

Silicates are the most common interferents in the PMB reaction and potentially present in water samples. The 3 min dip test was carried out with a range of silicate containing samples (100 – 1000 mg L^-1^), as well as with samples containing only silicate and no phosphate. These were compared to a blank sample to study whether false positives may occur.

**Figure S8.1**: Average intensity of blue colour formed after 3 min incubation of PAD in a range of sample solutions. A typical response for a 10 mg L^-1^ was compared directly with two samples containing silicate at 100 and 1000 mg L^-1^. This showed no significant difference between any samples investigated using ANOVA whereby F = 0.5589, F_crit_ = 3.467 at α = 0.05; n = 8. A comparison of a solution containing only 1000 mg L^-1^ silicate and a blank solution were also compared, again showing no significant differences. t_stat_ = 0.8584, t_crit_ = 2.145 at α = 0.05; n = 8 for two-tailed t-test.

It is well established that the formation of the molybdenum silicate complex favours longer reaction times. This would suggest that over the required short incubation period, no interference should be seen, however, over longer periods secondary reactions may occur. To investigate this, PADs were monitored for 30 min after initial water entry with an image captured every minute.

**(a)**

**(b)**B

**Figure S8.2**: Formation of the blue coloured molybdenum complex over time with solution of 10 mg L^-1^ phosphate with and without silicate present at 100 and 1000 mg L^-1^. This was also compared to the coloured complex formed when only silicates were present in solution at 1000 mg L^-1,^ n = 6. **(a)** Reaction occurring over 30 min showing the formation of the molybdenum-silicate complex was far slower than the molybdenum phosphate complex. **(b)** Reaction over 5 min, showing clearly within the window of 3 min that silicate does not interfere or give a false positive result.

A river water sample has a complex matrix potentially containing many ions which could interfere with a readout. To explore this, aliquots of a real river water sample were spiked with phosphate were compared to Milli-Q water spiked with the same concentrations (Figure S4.3). The water samples were taken from the Pocklington Canal (Yorkshire, UK, GPS location 53° 54' 4.2588'' N0° 48' 7.9164'' W), a water source with high calcium carbonate (100’s mg L^-1^) and chloride levels (up to 100 mg L^-1^) as well as many other water soluble ions at sub mg L^-1^ levels.

**(a)**

**(b)**B

**Figure S8.3**: **(a)** Comparison of colour intensity formed after 3 min incubation in a real water sample (Pocklington Canal, UK) and Milli-Q water, both were spiked with phosphate at 0 mg L^-1^, 2.5 mg L^-1^, 5 mg L^-1^, 7.5 mg L^-1^ and 10 mg L^-1^ (n = 8). **(b)** Comparison of colour intensity formed after 3 min incubation in a real water sample (Pocklington Canal, UK) and Milli-Q water, both were spiked with phosphate at 0 mg L^-1^, 1 mg L^-1^, 10 mg L^-1^, 100 mg L^-1^ and 1,000 mg L^-1^ (n = 8).

# 9. Field results

To account for water turbidity and false colour occurring from the water sample, negative control zones were added to the PAD. These two zones contained no reagents therefore the only colour produced here was due to the colour from the water sample (figure S9). A comparison of phosphate levels (PO_4_^3-^) determined using 3 min dip test when images were captured using either a laboratory-based method (flatbed scanner), or a field-based method (smartphone) (table S9).

**(a)**

**(b)**B

**Figure S9**: **(a)** Design of a sampling device with two negative control zones, to account for any colour from the water sample. **(b)** Examples of a result obtained from a volunteer showing the six reaction zones and two negative controls. Images were captured whilst devices were still with the container to ensure a simple procedure.

**Table S9** Phosphate concentrations obtained in water samples collected from the River Aire (Leeds, UK) and the Pocklington Canal (UK) captured using different either a flatbed scanner or a smartphone camera (n = 6).

| Water sample | Phosphate concentration /  mg L^-1^ | |
| --- | --- | --- |
|  | **Scanner** | **Camera phone** |
| River Aire (sample 1) | 2 ± 0.060 | 2 ± 0.200 |
| River Aire (sample 2) | 2 ± 0.060 | 2 ± 0.100 |
| Pocklington Canal | 0 ± 0.004 | 0 ± 0.009 |

During 2019 and 2020 a 16 km stretch of the Pocklington Canal was samples repeatedly, the data gathered from this can be collated and potentially be used to observe seasonal trends. This was achieved by plotting the results by season showing the percentage of results that were analysed yielding phosphate levels in each category. In total 103 samples were undertaken during this period, of which 97 yielded useful results.

**Figure S10**: Data collected via citizen-led sampling sessions across a 16 Km stretch of the Pocklington Canal during 2019 and 2020, plotted to show seasonal variation with continued sampling. The data is plotted to represent the percentage of devices categories at each phosphate level (none <1 mg L^-1^, low 1-3 mg L^-1^, medium 4-6 mg L^-1^ and high ≥ 7 mg L^-1^). n = 97.

# 10. Figure permissions

Figure 7 contains a map created using QGIS Desktop 3.16.10 software. This software is free and open source. QGIS do not hold copyrights on any images created using their software. As such the image in figure 7 is free of copyrights and can be freely shared.

Several images contain screen shots of the Riverdip software. The authors are the copyright holders for the software and thereby have the rights to publish these images.

# 11. References

1.	E. Carrilho, A. W. Martinez and G. M. Whitesides, *Anal. Chem.*, 2009, **81**, 7091-7095.

2.	Y. Lu, W. W. Shi, L. Jiang, J. H. Qin and B. C. Lin, *Electrophoresis*, 2009, **30**, 1497-1500.

3.	K. L. Peters, I. Corbin, L. M. Kaufman, K. Zreibe, L. Blanes and B. R. McCord, *Analytical Methods*, 2015, **7**, 63-70.

4.	K. E. Boehle, J. Gilliand, C. R. Wheeldon, A. Holder, J. A. Adkins, B. J. Geiss, E. P. Ryan and C. S. Henry, *Angew. Chem., Int. Ed.*, 2017, **56**, 6886-6890.

5.	R. B. Dean and W. J. Dixon, *Anal. Chem.*, 1951, **23**, 636-638.

6.	*American Public Health Association, American Water Works Association, Water Environment Federation*, 1999, 252.
